# The Beneficial Evaluation of the Healthy City Construction in China

**Published:** 2017-06

**Authors:** Yuming WANG, Xinxin WANG, Fangxia GUAN

**Affiliations:** 1. Henan Provincial People’s Hospital, Zhengzhou, Henan, P.R. China; 2. The First Affiliated Hospital of Zhengzhou University, Zhengzhou, Henan, P.R. China; 3. School of Life Science, Zhengzhou University, Zhengzhou, Henan, P.R. China

**Keywords:** Healthy city, Beneficial evaluation, China

## Abstract

**Background:**

Creating healthy cities promotes socio-economic development, thus, the creation of such cities has been receiving more attention from the Chinese government and the Chinese people. In the current study, the intention was to conduct a comprehensive evaluation of the creation of healthy cities in Henan province in central China.

**Methods:**

We randomly selected 18 cities (7 healthy cities and 11 non-healthy cities) in middle regions of China in 2013 and established evaluation indices to evaluate the beneficial effects by horizontal and vertical comparison analyses.

**Results:**

Creating the healthy cities promoted health service, economic development, spiritual and ecological success. This was achieved by constructing cities and changing work style among the officials and establishment of patriotic health organizations.

**Conclusion:**

This is the first comprehensive, in-depth, appraisal of the healthy cities in China. We suggest that creating the healthy cities should be promoted a larger extend world-wide since it is beneficial at many levels.

## Introduction

The concept of the healthy city was first promulgated by the World Trade Organization in 1988 (1). The World Health Organization (WHO) issued ten specific standards for the healthy city in 1996. These standards included providing for improved secure environment, nutrition, water, housing, health benefits and effective waste disposal systems. Another important aspect of the healthy city was to have groups of citizens assisting each other, and various organizations work in a coordinated manner to improve the health of the city. It was also important to have citizens of the city intimately involved in the formulation of policies that affect the health and welfare of daily life. Equally important is providing a place for entertainment and leisure activities that would enhance communication among the population. The preservation of cultural heritage and respecting diverse lifestyles and race and religious beliefs is another important aspect of the healthy city. These principles will lead to a healthy, disease-free life and increased longevity.

Approximately 1200 cities worldwide have participated in the healthy city project. In 1989, a campaign was launched to establish the national healthy city in China approved by the government, and assessing the success of the program was mandatory. Initially, there were only a limited number of cities examined in 4 rounds of inspection during the first 10 yr of the program. Since 1999, the process of determining the status of the national healthy city has been changed and now has a voluntary application, data review, field investigations, technological assessment, comprehensive review and finally making the public aware regarding the nomination of national healthy city (2, 3).

The basic demands of the standards of China’s national healthy city policy involves ten aspects including administration of patriotic health organizations, health education, city environment and sanitation, environmental protection, public and household drinking water sanitation, food security, prevention and treatment of communicable disease, prevention and treatment of vector-borne diseases, community sanitation, and the sanitation of the urban village and rural-urban continuum. The end of 2013, there were 178 cities that fulfilled the standard of the national healthy city, accounting for 24.8% of all the cities in China.

## Methods

In 2013, eighteen cities in middle region of China, Henan Province, a symbol of developing China, were randomly selected in the sample including 7 national healthy cities, and 11 non-national healthy cities. The effects of establishing healthy cities in China were evaluated in terms of health service and health level, economic development, spiritual civilization, ecological civilization, city construction, working style of government officers and construction of patriotic health organizations by means of field investigations, questionnaires, interviews, data reviews, horizontal and vertical comparison analyses.

## Results

### Promoting health service and improving health level

Statistical analysis revealed that the average life expectancy of the residents in healthy cities was 77.81±2.13 yr compared with 73.78± 3.23 yr in non-healthy cities, difference was statistically significant (*P*<0.05).

#### Disease prevention and control:

The incidence of infectious diseases in healthy cities was lower than that in non-healthy cities (*P*<0.05). In 2012, the morbidity in healthy cities was 369.12/100000 persons, compared to 527.74/100000 persons in non-healthy cities. National healthy cities made greater efforts to prevent and treat chronic diseases, usually with a comprehensive prevention and control strategies in demonstration areas. The rate of residents in healthy cities who engage in active exercise have a low-salt diet, quit smoking and alcohol intake control were 47.6%, 27.6%, and 13.2%, respectively, while in non-healthy cities it were 32.2%, 18.4% and 32.2%, respectively. There was a statistically significant difference (*P*<0.05). The proportion of residents with knowledge of chronic disease prevention and control as outstanding, good, ordinary, and poor levels were are 19.7%, 21.6%, 33.2% and 25.5%, respectively, while that in non-healthy cities were 8.2%, 11.4%, 37.6% and 42.8% respectively; there was a statistical significance in the level of outstanding, good and poor (*P*<0.05). Finally, national healthy cities have better institutions and organizations for disease prevention and control, better staffs, and more resources than non-healthy cities.

#### Health supervision:

Management with certificates in public, health system, registration system, supervision, and monitoring system were found in healthy cities. Those holding hygienic licenses and health certificates in national healthy cities were 96% and 85% in non-healthy cities. Additionally, the health supervision an institution in the national healthy cities had better construction, personnel, and funding.

#### Medical administration:

In the national healthy cities, the proportion of healthy institutions established with standardized managed infectious diseases departments or preview triage spot was 95%, however, only 80% in the national non-healthy cities. The national healthy cities enjoyed better hygienic conditions, and the hospital environment was greatly improved for the public.

#### Women’s community:

The community health services organization met the requirements of housing and personnel quality in national healthy cities. The tobacco control, healthy life, medical care, prevention and control of the occupational diseases were intensified in the public relations campaign. The ability of people to engage in self-care was improved. Finally, there were excellent institutions and networks for health education, personnel, and funding.

#### Health work in rural areas:

Infectious diseases and public health emergencies in the national healthy towns and villages were reported by the network directly. Triage spots for infectious diseases previewing were also widely established.

### Promoting the economic development

The economic growth grew significantly and the average gross national product (GNP) of national healthy cities in Henan province was 85.2 billion and 213.1 billion, respectively in 2006 and 2012. In non-healthy cities, it was 59.2 billion and 136.2 billion, less than in the national healthy cities. Vertical comparison in a city indicated that the economic growth rate was 16.2% before it was established as a healthy city, and it rose to 18.6% after being designated as a healthy city. One reason for the rapid economic growth in national healthy cities was the improvement of investment in environment, which was favorable for attracting business investment.

### Promoting the spiritual civilization

The establishment of a healthy city is the basis for the construction of the civilized city. Health is a most important factor in evaluating whether a city is civilized. There are 2 national healthy cities in Henan province, 4 nominated national healthy cities and 5 provincial civilized cities, all of which built into healthy cities before their nomination. The course of construction to healthy cities is the process of raising the level of modern management, improving civilization and hygienic quality in the city (4, 5). In 2011, the rate of awareness regarding health knowledge and healthy behavior in healthy cities was significantly higher than that in non-healthy cities. Awareness of health knowledge in healthy cities was 95.2%, while only 78.8% in non-healthy cities. In healthy cities, the smoking rate was 16.7%, alcohol addiction rate 7.6%, irregular diet rate 19.4%, irregular rest rate 8.7%, lack of physical exercise 35.2%, rate of people without knowledge of hand washing before eating or after defecation was 6.6%, while the proportion in “non-healthy” cities was 22.7%, 9.2%, 25.6%, 14.7%, 41.2% and 21.7% respectively. The difference was statistically significant (*P*<0.05).

### Promoting the level of ecological civilization

Environmental pollution control was improved in healthy cities. The rate of household garbage and centralized urban sewage processing to hazard-free were 96.27% and 96.96%, respectively. While the rate in non-healthy cities was 78.83% and 80.48%, that was significantly less than that in healthy cities. The effect of water quality protection was also greatly improved in national healthy cities. The water quality in centralized drinking water source was 100% of the national standards based on the environmental quality of surface water and groundwater quality standards. The greening level was also significantly improved. In national healthy cities, the percentage of greening coverage was 42.3%, greening rate was 38.4%, and green areas per capita in public parks were 10.12 m^2^. In contrast, the percentage was 35.82%, 33.33% and 7.84m^2^, respectively in national non-healthy cities. The difference was statistically significant (*P*<0.05).

### Promoting the construction of cities

The deficiencies of the city during the construction of healthy cities were significantly improved. Usually, the urban village, the rural-urban continuum, the back street and the farmer’s market were the most disorganized and dirty spaces in the cities making these cities more vulnerable to urban decay (6). The living environment of the people was greatly improved. For example, the rates of comprehensive management, the standard administration of the farmer’s market, and the asphalt road of the back street reached 95% in national healthy cities, while in national non-healthy cities it was significantly less. The infrastructure for environmental sanitation was markedly improved in healthy cities and the construction of healthy cities facilitated the establishment of corresponding standards and the implementation of regulations. The collaboration of relevant departments was also markedly improved.

### Promoting the transformation of cadre style

The construction of healthy cities promoted the active learning among the officials because it brought together many aspects including law, policies, and regulations (7,8). This dramatically changed the governmental working style. Establishment of healthy cities is a people benefit project, which calls for the officers to go into the population to do practical and good works for the masses (8). People experienced the changes, gained the benefits, and the relationship between governmental officials and the massesimproved. Constructing healthy cities improved the credibility of the government, promoted the satisfaction of the people to their health status. The rate of satisfaction in the national healthy cities was 92%, compared to 74% in non-healthy cities. City officials received training and practice during the process, which will also provide greater opportunity for promotion to higher office.

### Promoting the construction of patriotic health organizations

In 7 national healthy cities, there were total 198 employees who were working for patriotic health institutions, 28 persons in each city on average, while only total 71 staffs and 7 on average in 11 national non-healthy cities. Meanwhile, the funds, tasks, and responsibilities were further intensified and defined in the patriotic health offices of national healthy cities.

## Discussion

The construction of the healthy city in China began in 1980s, and at the same time, WHO advocated and launched the campaign of constructing the healthy cities. This was regarded as a global strategic goal. There are two main differences between the healthy city of China and WHO, one of which is the scope of the index system. The aim of healthy city in China is to govern the environment of dirty and disorderly cities and to raise the citizens’ awareness of civilization and health. There are 61 specific indicators to evaluate a healthy city in China, all of which are of clear quantitative requirements. In contrast, the concept of the healthy city of WHO is broader with greater importance given to the factors affecting sanitation and the health of the residents, and there are no strict regulations or requirements; the indicators are only instructive. In addition, healthy cities in China are subject to the review of application materials, unannounced visits, and field technology evaluation by the government organizations before being finally nominated as the healthy city by the country. It pays attention both to the process and the outcome. By contrast, WHO advocates and encourages each city to struggle for a healthy city, without evaluation or nomination. The primary manner of assessment is self-assessment and it only focuses on the process.

This is the first comprehensive evaluation since its inception of the healthy city construction 25 yr ago in China. The benefits are obvious at multiple levels (individual, community, organizational, political, economic, ecological, etc.), particularly in terms of strengthening the effects on health, local political administrative system and public policies.

Currently, a program by Chinese Government to control the environmental pollution and improve public health has been instituted. The importance and urgency of organizing the patriotic health campaign are redefined and more requirements that are stringent were put in place. Patriotic health legislation work, theoretical research and propaganda work, the rural areas’ creating work, self-construction and long-term management are all expected to be strengthened in the future.

Community participation and empowerment in the healthy cities program will be improved (9). Intersection coordination in the country and international collaboration in the world could further strengthen the project (4). Although there are similar reports in European countries (10, 11), evaluations on the healthy cities worldwide has not been fully implemented. This study provides important recommendations for creating healthy cities, especially in developing countries.

## Conclusion

Our investigation and evaluation of the healthy city construction in China shed light on the beneficial effects in terms of promoting health level, economic development spiritual and ecological civilization, city construction, cadre style, as well as patriotic health organizations. However, creating healthy cities remains a word-wide project that requires more participation and regulation to optimize its function.

## Ethical considerations

Ethical issues (Including plagiarism, informed consent, misconduct, data fabrication and/or falsification, double publication and/or submission, redundancy, etc.) have been completely observed by the authors.
